# Neoadjuvant chemotherapy prior to preoperative chemoradiation or radiation in rectal cancer: should we be more cautious?

**DOI:** 10.1038/sj.bjc.6602960

**Published:** 2006-02-07

**Authors:** R Glynne-Jones, J Grainger, M Harrison, P Ostler, A Makris

**Affiliations:** 1 Mount Vernon Cancer Centre, Northwood, Middlesex, HA6 2RN, UK

**Keywords:** rectal cancer, neoadjuvant chemotherapy, chemoradiation

## Abstract

Neoadjuvant chemotherapy (NACT) is a term originally used to describe the administration of chemotherapy preoperatively before surgery. The original rationale for administering NACT or so-called induction chemotherapy to shrink or downstage a locally advanced tumour, and thereby facilitate more effective local treatment with surgery or radiotherapy, has been extended with the introduction of more effective combinations of chemotherapy to include reducing the risks of metastatic disease. It seems logical that survival could be lengthened, or organ preservation rates increased in resectable tumours by NACT. In rectal cancer NACT is being increasingly used in locally advanced and nonmetastatic unresectable tumours. Randomised studies in advanced colorectal cancer show high response rates to combination cytotoxic therapy. This evidence of efficacy coupled with the introduction of novel molecular targeted therapies (such as Bevacizumab and Cetuximab), and long waiting times for radiotherapy have rekindled an interest in delivering NACT in locally advanced rectal cancer. In contrast, this enthusiasm is currently waning in other sites such as head and neck and nasopharynx cancer where traditionally NACT has been used. So, is NACT in rectal cancer a real advance or just history repeating itself? In this review, we aimed to explore the advantages and disadvantages of the separate approaches of neoadjuvant, concurrent and consolidation chemotherapy in locally advanced rectal cancer, drawing on theoretical principles, preclinical studies and clinical experience both in rectal cancer and other disease sites. Neoadjuvant chemotherapy may improve outcome in terms of disease-free or overall survival in selected groups in some disease sites, but this strategy has not been shown to be associated with better outcomes than postoperative adjuvant chemotherapy. In particular, there is insufficient data in rectal cancer. The evidence for benefit is strongest when NACT is administered before surgical resection. In contrast, the data in favour of NACT before radiation or chemoradiation (CRT) is inconclusive, despite the suggestion that response to induction chemotherapy can predict response to subsequent radiotherapy. The observation that spectacular responses to chemotherapy before radical radiotherapy did not result in improved survival, was noted 25 years ago. However, multiple trials in head and neck cancer, nasopharyngeal cancer, non-small-cell lung cancer, small-cell lung cancer and cervical cancer do not support the routine use of NACT either as an alternative, or as additional benefit to CRT. The addition of NACT does not appear to enhance local control over concurrent CRT or radiotherapy alone. Neoadjuvant chemotherapy before CRT or radiation should be used with caution, and only in the context of clinical trials. The evidence base suggests that concurrent CRT with early positioning of radiotherapy appears the best option for patients with locally advanced rectal cancer and in all disease sites where radiation is the primary local therapy.

The term neoadjuvant chemotherapy (NACT) was suggested over 20 years ago (Frei, 1982). Given that tumour size is a predictor of outcome in most disease sites, NACT has often been proposed with the aim or reducing the gross tumour burden before surgery, radiation or chemoradiation (CRT). The original hypothesis proposed that neoadjuvant cytotoxic chemotherapy could shrink a locally advanced tumour, and thereby allow more effective local therapy. Reducing the size of a tumour may facilitate a curative surgical resection. Also, in general, smaller cancers may be more oxygenated, and hence easier to control with radiation (Fletcher, 1980, 1984). Since then, the breadth of treatment possibilities has been extended as more effective combinations of cytotoxic chemotherapy and more recently new molecular targets that potentially reduce the risks of metastatic disease have been introduced.

In stages II and III of rectal cancer the optimal multimodality management remains an increasing challenge. Surgery remains the mainstay of treatment, but a high risk of local recurrence and poor survival (40–55% at 5 years) has been reported even for patients with mobile/resectable rectal cancer despite a curative resection and postoperative CRT (Tepper *et al*, 2002). Despite improved local surgical and radiotherapeutic techniques, locoregional relapse is now exceeded by the rate of development of systemic metastases. The Dutch rectal cancer study, CKVO 95–04, which randomised between surgery alone and short-course preoperative radiotherapy followed by immediate surgery, found local regional control improved from 91.6 to 97.6% at 2 years with preoperative radiotherapy (Kapiteijn *et al*, 2001). However, the rate of metastatic disease was 18% in both arms at 2 years. Despite low rates of local recurrence, 25–35% of patients with rectal cancer still eventually die from metastatic disease. In patients with unresectable disease (Frykholm *et al*, 2001) both local control and metastatic disease remain issues.

There is increasing enthusiasm for preoperative CRT in the management of rectal cancer. The advantage of using radiotherapy in the preoperative setting includes the potential to increase the likelihood of a R0 resection; to reduce the risk of tumour seeding; less acute toxicity; and enhanced radio-sensitivity because of better oxygenated cells as opposed to hypoxic cells in scar tissue; and an improved chance of sphincter preservation achieved by downstaging the tumour. Finally, compliance to treatment may be more easily achieved in the preoperative compared to the postoperative setting.

Recent data from studies in advanced/metastatic colorectal cancer show high response rates to combination cytotoxic chemotherapy (Cassata *et al*, 2001; Cassidy *et al*, 2004). Three drug combinations of 5-fluorouracil (5FU), oxaliplatin and irinotecan may now give even higher response rates in the region of 75% (Calvo, 2002; Falcone *et al*, 2002; Ychou *et al*, 2003). This evidence of efficacy coupled with the introduction of novel molecular targeted therapies (Saltz *et al*, 2002; Cho *et al*, 2003; Cunningham *et al*, 2004; Tabernero *et al*, 2004), has heightened an interest in delivering NACT in locally advanced rectal cancer. Where there are delays (as in the UK) in starting CRT or RT, because of a lack of resources, the use of NACT is appealing. It seems plausible that that NACT might compensate for these delays, and enhance both local and systemic control, halt further tumour progression in addition to reducing patient anxiety. However, there is no evidence to support this approach. In contrast, this new enthusiasm appears currently waning in other sites such as head and neck and nasopharynx cancer where traditionally NACT has been used (Garden, 2005).

With the aim of reducing systemic micrometastases, the use of neoadjuvant combination chemotherapy using oxaliplatin and 5FU before CRT has been explored in rectal cancer (Calvo *et al*, 2003; Chau *et al*, 2005). A Spanish study (Calvo *et al*, 2003) explored a short, intense induction regimen of oxaliplatin and 5FU (FOLFOX 4) before conventional CRT, which appeared to improve the tumour downstaging when compared to historical results in their unit previously achieved with CRT alone. This strategy of NACT claims to achieve a 29% complete pathological response rate in locally advanced rectal cancer. Studies from the UK have shown that similar neoadjuvant drug combinations in locally advanced rectal cancer can be delivered without apparently compromising future compliance to CRT (Chau *et al*, 2003, 2005).

Not all clinically localised cancers can be cured by radiation alone. Larger, more advanced cancers are more difficult to control locally than smaller ones and require higher doses of radiation (Fletcher, 1980; Perez *et al*, 1998). It is well recognised that factors such as hypoxia, which are often a consequence of rapid tumour growth have a negative impact on local control and survival from radiation therapy (Fyles *et al*, 1998; Nordsmark and Overgaard, 2000). Additionally, patients with more advanced tumours also are at a higher risk of systemic metastases. Hypoxia-driven cellular mechanisms may also contribute to a poor outcome, producing a more aggressive locoregional disease, and promotion of invasive capacity and angiogenesis (Walenta *et al*, 2000; Brizel *et al*, 2001). It has always been supposed that the anticancer effects of radiotherapy are mediated through oxygen associated free radical production (Hall, 2000). Others have stressed the importance of the overall time factor in combined modality treatment of head and neck cancer (Peters and Withers, 1997) in that accelerated repopulation can occur for any clonogens surviving a therapeutic intervention. Owing to this, novel treatment strategies have been devised. These aim to improve local and distant control. A number of biological rationales have been developed, including reducing the duration of potential maximum cellular proliferation by increasing the dose and intensity of treatment during the latter part of radiotherapy, or the addition of concurrent chemotherapy. An alternative strategy has been to use more intensive neoadjuvant or postoperative adjuvant chemotherapy. Consequently, combinations of sequential or concurrent chemotherapy and external beam irradiation have been in clinical use since the 1980s.

For the purpose of this article, chemotherapy should be considered as ‘neoadjuvant’ if it is administered as induction before radiotherapy, ‘concurrent’ if administered during the course of radiotherapy and ‘consolidation’ if delivered after radiation or CRT. In this review, we aim to explore the advantages and disadvantages of these separate approaches both in rectal cancer and other disease sites.

## MATERIALS AND METHODS

A literature search was performed using Medline and Cancerlit over the period 1996–2005 supplemented by hand searching of abstracts from the proceedings of the American Society of Clinical Oncology meetings to provide evidence for this discussion. Several keywords have been used which include synchronous, neoadjuvant, induction, concurrent, consolidation chemotherapy, CRT, radiotherapy and cancer.

### Neoadjuvant chemotherapy

#### Advantages

There are many theoretical advantages to NACT, which include the potential to eradicate distant micrometastases at an early stage in the evolution of the disease, utilization of the embryonic tumour blood supply and treatment of a fit patient, before, not after the depredations of surgery. In contrast, in the postoperative setting the patient is often unfit, and there is a significant chance of hypoxia within surgical scars or any residual disease. There is also evidence that the primary tumour without metastatic disease may be more sensitive to chemotherapy than metastatic lesions (Von Hoff *et al*, 1986; Maehara *et al*, 1990).

Tumour shrinkage with chemotherapy potentially allows improved tumour vascularity. Theoretically, the consequences of this are improved oxygenation and higher intratumoural levels of cytotoxic drugs – although there is only scanty data to confirm this hypothesis (Milas *et al*, 1995; Taghian *et al*, 2005). This is not just an issue of the tumour getting smaller, since it would also be expected that significant cell loss with cytotoxic chemotherapy may lead to a reduction in oxygen consumption (Milas *et al*, 1995). These factors of better oxygenation and improved drug access from a lower interstitial pressure (Taghian *et al*, 2005) in turn may enhance tumour sensitivity to chemotherapy or radiation. In addition, in the clinic, oncologists endorse the use of NACT to reduce the primary tumour volume and consequently reduce the high-dose radiation volume. This change in field size can allow avoidance of the tolerance dose for critical structures.

Randomised trials have established a well-defined role for NACT before surgical resection both to promote resectability and to allow more conservative surgery in primary breast cancer (Mauriac *et al*, 1991; Scholl *et al*, 1994; Makris *et al*, 1998; van der Hage *et al*, 2001; Bear *et al*, 2003). However, these trials in breast cancer have shown that there is no impact on survival whether the chemotherapy schedule is delivered before or after surgery. This strategy of NACT has also been used in head and neck cancer (The Department of Veterans Affairs Laryngeal Cancer Study Group, 1991; Lefevre *et al*, 1996), where it has resulted in significant downstaging of a locally advanced tumour, and allowed more patients to undergo a curative resection (Spaulding *et al*, 1994). However, the combination of NACT followed by radiotherapy was found to be inferior to concomitant CRT for organ preservation (Forrestiere *et al*, 2003).

There are several reasons that some researchers wish to deliver chemotherapy and radiotherapy at different times. First, the interaction between chemotherapy and radiotherapy often gives rise to such a marked increase in acute toxicity that it is necessary to reduce the dose and intensity of chemotherapy. Neoadjuvant chemotherapy may be associated with better compliance to treatment, and enable full systemic doses of chemotherapy to be delivered. In contrast, the effectiveness of adjuvant treatment in the postoperative setting has frequently been compromised by poor tolerance and marked dose reductions in oesophageal and rectal cancer.

NACT may act in some ways as a selection manoeuvre. There always exists a subset of patients with a very aggressive natural history, who are likely to fail to respond to chemotherapy. The delay before surgery means that patients who fail to respond locally, progress and may develop metastatic disease (Rohatgi *et al*, 2005) or deteriorate because of excess toxicity, can be selected out. These groups have no real possibility of benefit and can be spared the rigours of surgery or radical CRT.

A major advantage of NACT lies in the ability to observe and evaluate tumour clinical response during treatment. It is therefore often argued that response is a good testing ground for novel chemotherapy combinations, and that response to NACT can define good and bad prognostic groups – although these patients may well have a favourable outcome anyway. In contrast to crude measures like size on CT scans, the use of sequential positron emission tomography may define a major metabolic response to CRT (Guillem *et al*, 2004), and offers the opportunity to predict a better outcome in terms of overall survival. Failure to respond enables the delivery of a noncross resistant chemotherapy in breast cancer, as has been used in the Aberdeen neoadjuvant trial in breast cancer (Smith *et al*, 2002). However, in rectal cancer crossover studies at progression of first-line therapy have shown low rates of response to second-line chemotherapy (Tournigand *et al*, 2004).

#### Disadvantages

Induction chemotherapy will delay the definitive local treatment. Radiobiological evidence suggests that the potential doubling time of clonogenic cells can reach their maximum potential if cell loss is abolished (Kummermehr *et al*, 1992). Thus, by adding 9–12 weeks to the overall duration of treatment with 3–4 cycles of induction chemotherapy, tumour cell production could actually increase even if the volume of tumour on CT scanning appears less (Withers *et al*, 1988). Other authors have raised concerns regarding the time factor, and how preoperative chemotherapy may fail to take into account radiobiological principles of repopulation (Peters and Withers, 1997). NACT can accelerate the kinetics of tumour proliferation in residual cells, since it is observed that sequential responses from multiple lines of chemotherapy become shorter in duration.

As it is recognised that only a proportion of patients will respond to NACT, those who fail to respond in a large proportion of the cells are more likely to develop resistant cells. This process favours the selection of radio-resistant clones, and may reduce the efficacy of subsequent radiotherapy. In addition, if neo-adjuvant chemotherapy inhibits apoptotic pathways, then it may render what are normally apoptosis prone tumour cells less sensitive to radiation. It is also well recognised that chemotherapy may in some tumours upregulate epidermal growth factor receptor (EGFR) expression (De Pas *et al*, 2004), which in turn promotes angiogenesis, affects stromal proliferation, and may make the tumour more radio-resistant. Thus short-term response may not improve long-term outcome.

In cervical cancer, at least seven randomised trials have compared neoadjuvant cisplatin containing induction chemotherapy plus radiation therapy with radiation therapy alone (Chauvergne *et al*, 1990; Souhami *et al*, 1991; Tattersall *et al*, 1992; Kumar *et al*, 1994; Tattersall *et al*, 1995; Sundfor *et al*, 1996; Leborgne *et al*, 1997). These have consistently failed to show an improvement in overall or disease-free survival. In two trials (Souhami *et al*, 1991; Tattersall *et al*, 1992) results of NACT were significantly worse. It has been conjectured that this is a result of reducing compliance to the radiotherapy component and possibly enhancing accelerated repopulation of resistant clones with induction chemotherapy.

NACT especially in long courses (3–4 cycles) also may allow time for these drug-resistant clones to migrate from the primary site and seed distant or potential sanctuary sites. In small-cell lung cancer, where high responses to chemotherapy are observed, there is a significant increase in the incidence of brain metastases in patients receiving late compared to early thoracic radiotherapy, and a worse overall survival (Murray *et al*, 1993).

Finally, compliance to the CRT phase may be reduced if severe toxicity is experienced with NACT (Table 1).

### Concurrent chemotherapy

In rectal cancer there have been many different concurrent CRT schedules evaluated in phase I/II studies. Most researchers have used either a continuous infusion of 5FU (Rich *et al*, 1995), or the oral fluoro-pyrimidines (Hoff *et al*, 2000; Dunst *et al*, 2002; Kim *et al*, 2002; Dunst *et al*, 2004). Other more recent trials have integrated oxaliplatin into the CRT schedule (Rosenthal *et al*, 2002; Rodel *et al*, 2003; Glynne-Jones *et al*, 2004), and also irinotecan (Michell *et al*, 2001; Kennedy *et al*, 2004; Hofheinz *et al*, 2005).

The evidence base in terms of randomised trials of concurrent CRT in rectal cancer is limited. The North American standard of care in rectal cancer is to deliver postoperative chemotherapy and CRT to patients staged postoperatively as T3/4 or N+. This policy has been defined within the NIH consensus statement in 1990 based on the results of two pivotal randomised American studies (GTSG 7175 and NCCTG 79/47/51) (Gastrointestinal Tumor Study Group, 1985; Krook *et al*, 1991). The NSABP RO2 study (Wolmark *et al*, 2000), showed that the addition of radiotherapy produced significantly less locoregional failure as a first event (8 *vs* 13% at 5 years) compared to adjuvant chemotherapy alone. However, the addition of radiotherapy did not influence overall relapse-free or disease-free survival.

A further European randomised trial of postoperative 5FU-based CRT against surgery alone in Dukes B and C rectal cancer to a dose of 46 Gy demonstrated a significant improvement in local control, disease-free survival and overall survival for postoperative CRT (Tveit *et al*, 1997).

On the basis of this evidence, many mainland European countries have extrapolated the North American approach to the preoperative setting and deliver CRT to patients who are considered T3/4 or N+ on the basis of preoperative transrectal ultrasound. In France in 1994, a national consensus statement recommended the use of preoperative CRT in T3 and resectable T4 rectal cancer (Conference de consensus, Paris 1994).

A recent German phase III trial randomised 823 patients between preoperative CRT and postoperative CRT (patients received postoperative adjuvant chemotherapy in both arms of this trial). This study convincingly showed an improved therapeutic ratio for preoperative treatment. Local recurrence, acute and late toxicity were all statistically significantly reduced with the preoperative approach (Sauer *et al*, 2004). There was, however, no difference in the distant metastases rate or overall survival.

Two recent large trials have (from the EORTC and FFCD groups), have compared preoperative radiotherapy alone with preoperative CRT (Bosset *et al*, 2005a, 2005b; Gerard *et al*, 2005). The former trial has also used a second randomisation to compare postoperative adjuvant 5FU chemotherapy following CRT *vs* no further treatment (Bosset *et al*, 2005a). Both trials demonstrated a highly signigficant reduction in the risks of local recurrence, but showed no impact on metastases, disease-free survival or overall survival.

In addition two small randomised trials of CRT in the postoperative adjuvant setting also support the view that concurrent chemotherapy is the most effective strategy (Fountzilas *et al*, 1999; Cafiero *et al*, 2003). The first paper questions the additional advantage to consolidation chemotherapy following the main strategy of concurrent CRT. In contrast the data from Cafiero suggest that the beneficial results of concurrent CRT may not apply if these modalities are administered sequentially rather than concurrently.

#### Advantages

Prolongation of overall radiotherapy time allows accelerated repopulation. Concurrent CRT schedules therefore appear more efficient and may well offer more advantage in terms of locoregional control if our current hypotheses regarding accelerated tumour cell proliferation are correct. It is recognised that the rate of tumour growth is slower than the potential doubling time of tumour cells reflecting the cell loss factor. Cell production rates can be approximately 10 times faster than tumour growth rates (Trott, 1999). Repopulation is probably accounted for both by a decrease in the cell loss factor and in an increase in the rate of proliferation of surviving tumour cells (Fowler, 1991). Many consider that the increase in cellular repopulation relates both to increase in tumour oxygen and also cellular density changes. The advantage of the concurrent administration of radiotherapy and chemotherapy is that it may prevent the tumour repopulating.

In non-small-cell lung cancer (NSCLC), randomised studies comparing sequential *vs* concurrent treatment have shown an advantage to the concurrent approach; perhaps not surprisingly, there is an associated greater toxicity. Both Curran *et al* (2000) and Furuse *et al* (1999), as well as a recent preliminary report (Choy *et al*, 2002), have shown an advantage to early CRT followed by consolidation chemotherapy in terms of median survival over both induction followed by concurrent and sequential chemotherapy and radiotherapy. Individual trials have also demonstrated an overall survival benefit for concurrent CRT over sequential chemotherapy and radiation or radiation alone in nasopharyngeal cancer (NPC) of about 30% (Al-Sarraf *et al*, 1998; Chan *et al*, 2002; Lin *et al*, 2003).

A recently reported phase III trial (Forrestiere *et al*, 2003) randomised patients with locally advanced laryngeal cancer into three study groups. The first group received induction chemotherapy with cisplatin plus 5FU 120 h infusion every 3 weeks for 2–3 courses depending on response followed by radical radiotherapy. The second group received radiotherapy with concurrent administration of cisplatin at a dose of 100 mg m^−2^ on days 1, 22 and 43 but without any induction chemotherapy. The third group received radiotherapy alone. A total of 540 patients were randomly assigned to one of these three groups. Locoregional control was significantly better with concurrent cisplatin and radiotherapy (78 *vs* 61% for induction chemotherapy followed by radiotherapy). In addition 91% of patients who received induction chemotherapy were metastasis-free compared to 92% of those who received concurrent chemotherapy and radiotherapy. However, overall survival rates were similar in all three groups. The study concludes that radiotherapy with concurrent cisplatin should be the standard of care for patients who wish to preserve their larynx. The routine use of induction chemotherapy followed by radiotherapy in head and neck cancer is therefore not supported by the results of this trial.

Concurrent chemotherapy potentially introduces systemic doses of chemotherapy early at the same time as the intensive treatment of the primary tumour. Early chemotherapy will hopefully stop repopulation and downregulate any angiogenic mechanisms, which in turn may help to prevent metastases. There is an obvious potential that greater toxicity will result from concurrent chemotherapy.

#### Disadvantages

Concurrent chemotherapy may be associated with a high risk of enhanced acute toxicity, in part because chemotherapy may inhibit the ability of normal tissue to repair the sublethal radiation induced damage. In order to avoid excess toxicity, most clinicians have resorted to lower doses of chemotherapy and a maximum of two drugs. However, lower doses of chemotherapy imply less systemic effects on reducing metastatic disease. An alternative strategy is to reduce the total dose of radiotherapy, although this practice too has been criticised on the grounds of delivering an inadequate dose. Concurrent chemotherapy and radiotherapy may be associated with more acute and dose limiting toxicity, and also more late toxicity (Table 2).

### Consolidation chemotherapy

A third strategy (consolidation chemotherapy) is to deliver chemotherapy late in the process of radiotherapy or shortly after its completion. The role of using a consolidation chemotherapy schedule is currently being explored in the United Kingdom ACT II trial in anal cancer where patients are randomised to two courses of 5FU/cisplatin following the completion of CRT or no further treatment (James and Meadows, 2003). This policy is also likely to be explored in rectal cancer in a planned American ACOSOG study.

#### Advantages

Shrinking the tumour with radiotherapy may allow improved tumour vascularity and facilitate high levels of cytotoxic drugs reaching any remaining tumour cells. It is also possible that the small percentage of clonogenic tumour cells that manage to survive to the end of radiotherapy may be partially damaged but more actively proliferating and may have increased susceptibility to cytotoxic chemotherapy. In addition the high rate of failure in terms of distant metastases, suggests that residual malignant cells either in the primary site or elsewhere may require more effective and additional systemic methods of elimination if cure is to be achieved.

#### Disadvantages

The main disadvantage for administering systemically active chemotherapy in a consolidation phase is that this strategy does not allow a response to be assessed in terms of chemotherapy, and makes it difficult to decide later whether further chemotherapy is required. In addition, toxicity from CRT may compromise the dose intensity of consolidation chemotherapy.

A study from South Korea (Lee *et al*, 2002) enrolled patients with Stages II and Stage III rectal cancer following TME surgery. This study aimed to investigate whether early, as opposed to late, postoperative radiotherapy with chemotherapy was the optimal sequence. Radiotherapy started on Day 1 on the first cycle of chemotherapy in the early RT group and Day 1 of the third cycle of chemotherapy in the late RT arm. With a median follow-up of 37 months, 61 (22%) of the 274 eligible patients had experienced a recurrence of their rectal cancer, 23 in the early radiotherapy group *vs* 38 in the late radiotherapy group. The 4-year rate of disease-free survival was 81% for early radiotherapy and 71% for late radiotherapy. Both distant metastases and local regional recurrence appeared to show a trend towards being less frequent for early radiotherapy (15 *vs* 22% and 2 *vs* 6%, respectively). However, the 4-year overall survival was similar in both groups (84 *vs* 82%). This study seems to confirm the hypothesis that early radiotherapy may be advantageous, and mirrors data suggesting an improved outcome is obtained from early radiotherapy in studies of small-cell lung cancer and NPC (Table 3).

## DISCUSSION

The use of NACT before CRT in other disease sites remains controversial. Four meta-analyses have examined data from the published phase III trials in head and neck cancer, and all have concluded that concurrent rather than neoadjuvant induction chemotherapy regimens lead to improved survival (Stell, 1992; Munro, 1995; El-Sayed and Nelson, 1996; Pignon *et al*, 2000). The benefit appears to be 7% at 2 years and 8% at 5 years. Two of the meta-analyses (El-Sayed and Nelson, 1996; Pignon *et al*, 2000) showed no survival advantage to NACT.

Despite the enrolment of large numbers of patients with head and neck cancer in studies testing chemotherapy in the induction setting, and concurrently with radiation, only synchronous CRT has changed outcomes. There are no data to suggest that induction chemotherapy improves local control (Forrestiere *et al*, 2003). Neoadjuvant induction chemotherapy has shown a benefit in terms of organ preservation in head and neck cancer, but not in terms of overall survival. It remains unclear whether organ preservation is achieved by effective downstaging of the tumour or by selecting patients who are potentially curable by radiation alone. This is not necessarily the same as induction chemotherapy being able to predict the response to subsequent radiotherapy. The routine use of induction chemotherapy followed by radiotherapy in head and neck cancer is therefore not supported by the results of trials.

There is evidence that NACT may improve outcome in terms of disease-free or overall survival is strongest when NACT is administered before surgical resection (Law *et al*, 1997; MRC, 2002; Allum *et al*, 2003); although three metanalyses in oesophageal cancer (Malthaner and Fenlon, 2001; Urschel *et al*, 2002; Malthaner *et al*, 2004) failed to show a survival benefit for NACT before surgery. Neoadjuvant chemotherapy did not change the number or pattern of local distant or regional recurrence. This advantage does not appear to extend to all patients. Some subsets of patients with an excellent clinical (and pathological) response to NACT appear to have an extended survival time, but despite this observation, overall survival time of the whole group has usually failed to improve.

In NSCLC, the CALGB group established that two cycles of induction chemotherapy using cisplatin and vinblastine followed by radiotherapy to the chest increases median survival time when compared to radiotherapy alone. (Dillman *et al*, 1996; Sause *et al*, 2000). However, although induction chemotherapy reduced the rates of systemic failure, it has failed to improve local control. A meta-analysis has shown an advantage for cisplatin-based induction chemotherapy followed by radiotherapy over radiotherapy alone (Gordon and Vokes, 1999) but an advantage for NACT followed by CRT over CRT alone has not been observed. When the two strategies have been directly compared, concurrent chemotherapy and radiotherapy appears statistically superior to induction chemotherapy although at the price of more severe acute toxicity (Furuse *et al*, 1999; Curran *et al*, 2000, 2003).

In bladder cancer, it is possible that there is a small advantage for NACT before cystectomy (Grossman *et al*, 2003). However, in muscle invasive bladder cancer, the only trial that has compared neoadjuvant and concurrent CRT (RTOG 89-03) has not shown benefit in survival (Shipley *et al*, 1998).

Ian Tannock's first law of chemotherapy states: ‘Tumour response is not an endpoint of patient benefit’ (Tannock *et al*, 1996). There is no evidence that increasing the rate of a clinical or pathological complete response consistently correlates with an increase in disease-free and overall survival. By adding 12 weeks to the overall duration of treatment with 3–4 cycles of induction chemotherapy, tumour cell production could actually increase even if the volume of tumour on CT scanning appears less. Resistance to chemotherapy probably occurs earlier than evidence of tumour progression would lead us to believe (Smith *et al*, 2002). If NACT is administered before CRT, it may be appropriate to consider using a noncross resistant combination rather than the same drug, for example, oxaliplatin in both schedules. This strategy might overcome the limit of resistant clones developing during concurrent CRT.

In unresectable rectal cancer, or borderline resectable cancers where the circumferential margin is unsafe on preoperative MRI, the most important component is concurrent CRT. In contrast, NACT before surgery for resectable cancers might reduce the risk of subsequent metastatic disease, and is not so concerned with local control. Hence, this is a suitable question to be addressed in future research programmes if surgery alone is the primary treatment. Therefore, we need better initial patient selection, and we need a quick, valid and reliable method of assessing metabolic response or – perhaps better still – resistance to chemotherapy.

For locally advanced rectal cancer, current evidence suggests that preoperative CRT provides optimal locoregional control. However, distant failure remains common. The role of adjuvant chemotherapy either before or following CRT in improving disease-free or overall survival has yet to be established, despite evidence that response to induction chemotherapy may predict response to subsequent radiotherapy.

However, experience from other sites (multiple trials in head and neck cancer, NPC, NSCLC, small-cell lung cancer, and cervical cancer) does not support the routine use of NACT either as an alternative, or as additional benefit to CRT. Neoadjuvant chemotherapy does not appear to enhance local control over concurrent CRT or radiotherapy alone. Although there are some data to suggest a reduction in the risk of metastatic disease, the majority of these trials show an advantage for the combination of concurrent chemotherapy and radiotherapy rather than the sequential or induction approach.

## CONCLUSIONS

Our conclusion is that concurrent CRT with early positioning of radiotherapy therefore appears the best option in all disease sites where radiation is the primary local therapy. Neoadjuvant chemotherapy before CRT or radiation in rectal cancer should be used with caution, and only in the context of clinical trials. If NACT is used in this group of patients, a noncross resistant combination is recommended rather than the same drug, for example, oxaliplatin in both schedules. Also the observation of major clinical and pathological downstaging should *not* obviate the requirement for CRT either pre operatively or post operatively as there will remain a significant risk of local recurrence.

## Figures and Tables

**Table 1:** Neoadjuvant chemotherapy

**Potential advantages**	**Potential disadvantages**
Early treatment of micrometastases	Increase in duration of chemotherapy
Tumour will have intact blood supply	Delays definitive treatment
Delivery of full systemic doses	Favours development of resistant clones
Better compliance to chemotherapy treatment	May select radio-resistant clones
May enhance oxygenation and radio-responsiveness	May allow distant/sanctuary site seeding
May allow radical RT if tumour shrinks	May reduce compliance to chemoradiation
Potential for organ sparing if downstaged	
Potential for curative resection if downstaged	
Avoids surgery for resistant/rapidly progressive tumours	
Response to chemo predictive of response to radiation	
Response may define good/bad prognostic groups	

**Table 2:** Concurrent chemotherapy (chemoradiation – CRT)

**Potential advantages**	**Potential disadvantages**
Early treatment of micrometastases	Delivery of less than full systemic doses
May prevent repopulation during radiotherapy	Worse compliance to chemotherapy treatment
Tumour will have intact blood supply	More acute toxicity?
Potential for organ sparing if downstaged	More late toxicity?
Potential for curative resection if downstaged	
Avoids surgery for resistant/progressive tumours	
Response may define good/bad prognostic groups	
Trials show improved local control	
Trials show reduced rates of metastatic disease	

**Table 3:** Consolidation chemotherapy in addition to chemoradiation (CRT)

**Potential advantages**	**Potential disadvantages**
Early treatment of micrometastases	Does not allow assessment of response to chemo
May prevent repopulation during radiotherapy	Worse compliance to chemotherapy treatment
Tumour will have intact blood supply	Delivery of less than full systemic doses?
May enhance oxygenation and chemo-responsiveness	More acute toxicity?
Delivery of full systemic doses?	More late toxicity?
Cells may be more sensitive to chemotherapy	
Potential for organ sparing if downstaged	
Potential for curative resection if downstaged	
Avoids surgery for resistant/progressive tumours	
Response may define good/bad prognostic groups	
Trials show improved local control from CRT	
Trials show reduced rates of metastatic disease	
Trials show early radiotherapy is more effective	
